# Identification of Active Anti-Inflammatory Compounds in Sweet Potato Storage Roots Extracted with Ethanol via LC-Q-TOF-MS

**DOI:** 10.3390/molecules31030456

**Published:** 2026-01-28

**Authors:** Ryohei Sakuraoka, Hirofumi Masutomi, Katsuyuki Ishihara

**Affiliations:** Research and Development Division, Calbee, Inc., Utsunomiya 321-3231, Tochigi, Japan; r_sakuraoka@calbee.co.jp (R.S.); k_ishihara@calbee.co.jp (K.I.)

**Keywords:** anti-inflammatory effect, sweet potato, phytosterols, LC-Q-TOF-MS, MS-FINDER

## Abstract

Sweet potatoes (*Ipomoea batatas* (L.) Lam.) are known for their anti-inflammatory effects, which are attributed to their phytochemical content. Our previous study revealed that ethanol extracts of sweet potato storage roots (SP-EtOH-Ex) inhibit interleukin-6 (IL-6) production in RAW264.7 cells stimulated with lipopolysaccharide (LPS). However, the causative compounds responsible for the anti-inflammatory effect have not yet been identified. This study aims to identify the compounds responsible for the anti-inflammatory effect of SP-EtOH-Ex using liquid chromatography–quadrupole time-of-flight mass spectrometry (LC-Q-TOF-MS). The unknown compounds were measured using the auto MS/MS mode (data-dependent acquisition; DDA) of LC-Q-TOF-MS, and the resulting data were analyzed using MS-DIAL and MS-FINDER and also compared with those of the corresponding reference standards in terms of retention time and fragment ions. As a result, β-sitosterol (2.527–4.850 µg/mL), campesterol (75.74–93.63 ng/mL), and lauroyl diethanolamide (4.568–9.260 ng/mL) were identified and quantified in SP-EtOH-Ex. Moreover, the anti-inflammatory effect of these three compounds against RAW264.7 cells was investigated at varying concentrations of β-sitosterol (1 µg/mL, 5 µg/mL, 10 µg/mL), campesterol (10 ng/mL, 100 ng/mL, 1000 ng/mL), and lauroyl diethanolamide (1 ng/mL, 10 ng/mL, 100 ng/mL). The phytosterols β-sitosterol and campesterol suppressed LPS-induced IL-6 production at concentrations comparable to those present in SP-EtOH-Ex. In contrast, lauroyl diethanolamide did not similarly suppress LPS-induced IL-6 production. These results suggest that β-sitosterol and campesterol in sweet potato storage roots contribute to their anti-inflammatory effects. The lack of activity in lauroyl diethanolamide further supports that phytosterols are the primary anti-inflammatory constituents. The edible portion of sweet potatoes holds promise as a promising raw material with anti-inflammatory properties.

## 1. Introduction

Sweet potato (*Ipomoea batatas* (L.) Lam.) is a perennial herbaceous species of the family Convolvulaceae. It exhibits strong drought tolerance and can produce high yields even in nutrient-poor soils. Although global production is concentrated in China and regions of Africa, its storage roots are consumed worldwide and are recognized as nutrient-dense foods rich in dietary fiber, vitamins, and minerals, thereby offering potential health benefits [1]. Sweet potatoes are also notable for their abundant contents of phytochemicals, including carotenoids and anthocyanins [2,3]. Accumulating evidence has highlighted their relevance in immune modulation [4,5,6]. For instance, anthocyanins derived from purple-fleshed sweet potatoes suppress lipopolysaccharide (LPS)-induced production of pro-inflammatory mediators, including tumor necrosis factor-α (TNFα), interleukin-6 (IL-6), and nitric oxide (NO), in RAW264.7 macrophages and attenuate the proliferation of several cancer cell lines [4]. Likewise, the β-carotene present in orange-fleshed sweet potatoes has been reported to inhibit LPS-induced IL-6, NO, and prostaglandin E_2_ (PGE_2_) production in the same cell model [5]. Our previous findings demonstrated that we fractionated the ethanol extract of sweet potato storage roots using reversed-phase chromatography and found that the fraction eluting between 51 and 60 min significantly suppressed LPS-induced IL-6 production in RAW264.7 macrophages, whereas the other fractions showed little or no activity [7]. Based on these findings, the present study focused specifically on this bioactive fraction, rather than the entire extract, to identify the constituents responsible for its anti-inflammatory effects. For clarity, this fraction is referred to as SP-EtOH-Ex throughout the manuscript. Mass spectrometry has become indispensable in chemical biology research—including metabolomics, biosynthetic pathway analysis, foodomics, and exposomics—and remains one of the most powerful analytical platforms for characterizing low-molecular-weight natural products [8,9,10,11]. Numerous studies have proposed workflows for the structural elucidation of unknown compounds using high-resolution mass spectrometry [12,13,14,15]. In plant metabolite profiling, such efforts have predominantly focused on phenolic compounds, and many earlier studies relied heavily on manual interpretation of spectra with reference to the literature or spectral databases [16,17,18,19]. More recently, data-processing platforms such as MS-DIAL (RIKEN Center for Sustainable Resource Science, Kanagawa, Japan) have been developed to perform peak detection, deconvolution, and preliminary annotation of features from non-targeted LC–MS datasets in a systematic and reproducible manner. Annotation of unknown features typically involves matching experimentally acquired product-ion spectra with curated reference data. A range of computational tools—such as MS-FINDER, CSI:FingerID, CFM-ID, and MetFrag—enable in silico fragmentation and structural prediction, and their combined application has been shown to substantially improve identification accuracy [20,21,22,23,24].

The present study aimed to identify constituents within the ethanol extract of sweet potato storage roots that contribute to their anti-inflammatory effects using an integrated mass spectrometry-based approach.

## 2. Results

### 2.1. Annotation of Nine Compounds from the Ethanol Extract of Sweet Potato Storage Roots

All sweet potato samples used for SP-EtOH-Ex preparation were of the cultivar Beniharuka. Processing the raw LC-MS data of ethanol extracts of sweet potato storage roots (SP-EtOH-Ex) using MS-DIAL yielded a total of 18,863 detected peaks. Subsequent annotation by MS-FINDER (RIKEN Center for Sustainable Resource Science, Kanagawa, Japan) resulted in 7439 putative compounds. Among these, database filtering using FooDB and KNApSAcK narrowed the annotation candidates to gibberellin A3, cinnamic acid, N-hexadecanoylpyrrolidine, lauroyl diethanolamide, β-sitosterol, campesterol, coniferyl aldehyde, mellein, and cycloartenol.

**Figure 1:**
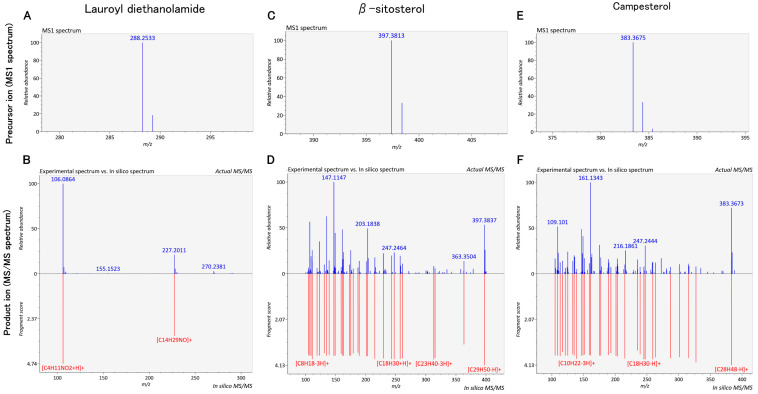
Precursor ions and product ions of compounds that ranked highly in both MS-FINDER and MassBank. These three compounds correspond to Level 1 according to Schymanski et al.’s criteria, indicating reliable identification. The compounds are annotated as lauroyl diethanolamide (**A**,**B**), β-sitosterol (**C**,**D**), and campesterol (**E**,**F**).

Ranking of these candidates using MS-FINDER and MassBank further refined their likelihood. In MS-FINDER, β-sitosterol, campesterol, coniferyl aldehyde, gibberellin A3, and cinnamic acid were ranked first; lauroyl diethanolamide and N-hexadecanoylpyrrolidine ranked between first and second; mellein ranked between second and fortieth; and cycloartenol ranked between third and eighth (Table S3). In MassBank, lauroyl diethanolamide, β-sitosterol, and campesterol ranked 1st, while coniferyl aldehyde ranked between 39th and 41st; the remaining compounds were not ranked (Table S3). Although some compounds did not rank in MassBank, their high MS-FINDER ranks supported the inclusion of these nine compounds as candidates potentially contributing to the anti-inflammatory activity of SP-EtOH-Ex (Figure 1 and Figure S1).

### 2.2. Identification and Quantification of Compounds in Sweet Potato Ethanol Extracts

**Figure 2:**
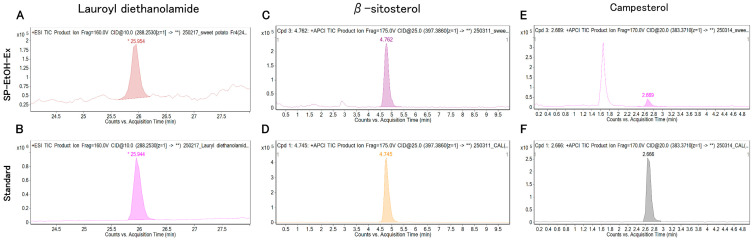
Compound chromatograms (CCs) of SP-EtOH-Ex corresponding to compounds meeting Level 1 of Schymanski et al.’s identification criteria based on standard reference compounds, and CCs of each standard reference compound. Lauroyl diethanolamide (**A**,**B**), β-sitosterol (**C**,**D**), campesterol (**E**,**F**). SP-EtOH-Ex: ethanol extracts of sweet potato storage roots. An asterisk (*) shown in (**A**,**B**) denotes manually integrated peaks. The double asterisk (**) shown in (**A**–**F**) indicates that the MS/MS chromatograms represent the full range of product ions obtained for the specified precursor ion.

**Figure 3:**
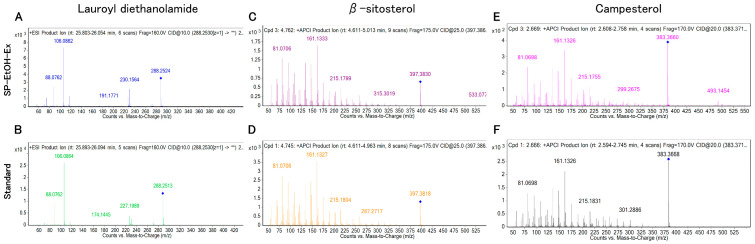
SP-EtOH-Ex product ions and product ions of each reference standard. Lauroyl diethanolamide (**A**,**B**), β-sitosterol (**C**,**D**), campesterol (**E**,**F**). These three compounds corresponded to Level 1 of Schymanski et al.’s identification criteria based on standard compound identification. SP-EtOH-Ex: ethanol extract of sweet potato storage roots. The blue diamond indicates the precursor ion selected for fragmentation in the MS/MS analysis. The double asterisk (**) indicates that the MS/MS chromatograms represent the full range of product ions obtained for the specified precursor ion.

The annotation confidence of the nine candidate compounds was evaluated by comparing retention times (RTs), precursor ions, and product-ion masses between SP-EtOH-Ex and authentic standards. Representative compound chromatograms (CCs) for standards and SP-EtOH-Ex are shown in Figure 2A–F and Figure S2A–H, and product-ion spectra are shown in Figure 3A–F and Figure S3A–H. The differences in RTs between standards and SP-EtOH-Ex were as follows: lauroyl diethanolamide, −0.04 to 0.02 min; β-sitosterol, −0.028 to 0.051 min; campesterol, −0.003 to 0.017 min; N-hexadecanoylpyrrolidine, −0.278 to 0.258 min; coniferyl aldehyde, 20.724 min; mellein, 6.215 to 6.453 min; and cycloartenol, 0.631 to 0.712 min (Table 1 and Table S4). Among these, lauroyl diethanolamide, β-sitosterol, and campesterol met the retention-time criterion of ±0.25 min proposed by Pérez-Ortega et al., demonstrating sufficient RT consistency with their corresponding standards [15]. The relative mass errors were 0.69–7.98 ppm for lauroyl diethanolamide, −4.03–2.26 ppm for β-sitosterol, −1.57–6.26 ppm for campesterol, −1.29–5.48 ppm for N-hexadecanoylpyrrolidine, −464.62 ppm for coniferyl aldehyde, 19.55–37.42 ppm for mellein, and −7.82–12.21 ppm for cycloartenol. Under the same analytical conditions as those used for annotation, gibberellin A3 and cinnamic acid were not detected (signal-to-noise ratio (S/N) < 3). Absolute quantification using calibration curves for each standard revealed that SP-EtOH-Ex 1–3 contained lauroyl diethanolamide (4.568–9.260 ng/mL), β-sitosterol (2.527–4.850 µg/mL), and campesterol (75.74–93.63 ng/mL) (Table 2).

### 2.3. β-Sitosterol and Campesterol in Sweet Potato Roots Contribute to Anti-Inflammatory Activity

**Figure 4:**
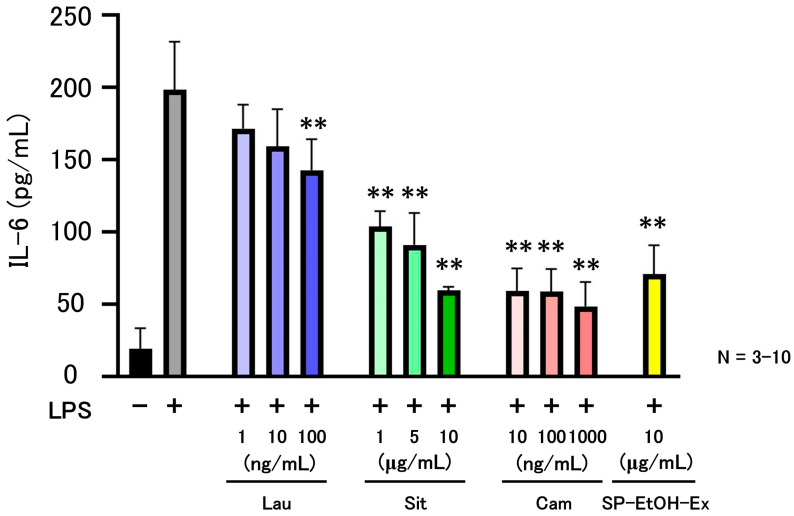
Measurement of the pro-inflammatory IL-6 24 h after LPS stimulation in the presence of lauroyl diethanolamide, β-sitosterol, campesterol, and SP-EtOH-Ex. Concentrations of Lau (lauroyl diethanolamide), Sit (β-sitosterol), and Cam (campesterol) are indicated on the x-axis (ng/mL or μg/mL). Mean ± standard error of the mean (SEM). SP-EtOH-Ex values indicate sample number. One-way ANOVA with Tukey’s post hoc test; **: *p* < 0.01; IL-6: interleukin-6; LPS: lipopolysaccharide; Lau: lauroyl diethanolamide; Sit: β-sitosterol; Cam: campesterol; SP-EtOH-Ex: ethanol extract of sweet potato roots.

Measurement of IL-6 concentrations in culture supernatants 24 h after LPS stimulation showed that SP-EtOH-Ex significantly suppressed IL-6 secretion compared with the LPS-only control (Figure 4). SP-EtOH-Ex 1 produced the greatest reduction, followed by SP-EtOH-Ex 2 and SP-EtOH-Ex 3. β-Sitosterol suppressed IL-6 production at concentrations comparable to those present in SP-EtOH-Ex (2.527–4.850 µg/mL). Similarly, campesterol suppressed IL-6 production at concentrations comparable to those in SP-EtOH-Ex (75.74–93.63 ng/mL) (Figure 4). In contrast, lauroyl diethanolamide did not suppress IL-6 production at concentrations found in SP-EtOH-Ex (4.568–9.260 ng/mL).

## 3. Discussion

In this study, we sought to annotate and identify the anti-inflammatory constituents present in an ethanol extract of sweet potato storage roots. As a result, nine compounds were annotated by MS-FINDER, among which lauroyl diethanolamide, β-sitosterol, and campesterol were successfully identified. Of these, β-sitosterol and campesterol exhibited anti-inflammatory activity at concentrations corresponding to those present in the ethanol extract of sweet potato storage roots.

The choice of mass spectrometric techniques is critical for the identification of unknown compounds. Kind et al. reported that high-resolution mass spectrometry (HRMS) used in non-target screening typically provides resolving powers of 10,000–450,000 (FWHM) and mass accuracies of <1–5 ppm [25]. Sasse et al. also emphasized that non-targeted analyses require a minimum resolving power of 10,000 and mass errors below 10 ppm [26]. The Agilent 6530 Q-TOF mass spectrometer used in the present study has a maximum resolving power of 20,000 (FWHM) and exhibits a MS1 mass accuracy better than 2 ppm. Therefore, this instrument meets the requirements for identifying unknown compounds in complex matrices.

Numerous workflows for the identification of unknown compounds have been reported [13,14,15,21,27,28,29,30]. MS-FINDER predicts plausible molecular structures via in silico fragmentation of candidate formulas derived from exact mass, isotopic patterns, and product-ion information. These candidate structures are ranked using multiple scoring components, including hydrogen rearrangement rules, with reference to 23 built-in databases [20]. MassBank, the first public mass spectral repository for small molecules (<3000 Da) in the life sciences, is one of the most widely used spectral databases for structural elucidation [31]. Similar to GNPS and NIST [32], MassBank enables annotation by matching experimentally obtained product ions to those in standard spectral libraries [33]. Although MS-FINDER exhibits high predictive accuracy compared to existing in silico fragmentation tools, perfect identification cannot be expected [21,34,35]. Mallmann et al. reported that MS-FINDER alone correctly identified 70% of tested compounds [34], whereas Vaniya et al. reported 53% accuracy [21]; however, integrating publicly available databases such as NIST [32], METLIN [36], and MassBank of North America [37] data increased the identification accuracy to 78%. These findings highlight the importance of using multiple tools to enhance the identification accuracy.

In this study, MS-FINDER and MassBank were used to annotate nine potential anti-inflammatory constituents in sweet potato ethanol extracts. For small molecules, Schymanski et al. proposed a five-level confidence scale for compound identification, wherein Level 1—the highest confidence—is achieved when the retention time, precursor ion, and product ions match those of an authentic standard [38]. Sasse et al. recommended targeting at least Level 2 confidence for metabolite annotation in complex samples [26], while, regarding retention time (RT), Pérez-Ortega et al. suggested an optimal window of ±0.25 min [15]. Chaleckis et al. described a tiered workflow for evaluating annotation confidence, noting that the ideal RT tolerance varies with chromatographic mode; for the RP column used in this study, ±0.1 min is recommended [14]. In a study screening more than 630 multi-residue food contaminants using the same Agilent 6530 Q-TOF instrument, Pérez-Ortega et al. reported relative mass errors between −5.51 and 7.94 ppm [15].

When comparing RTs and precursor ions between the annotated compounds and SP-EtOH-Ex 1–3, only lauroyl diethanolamide, β-sitosterol, and campesterol satisfied these criteria. Their product-ion spectra were also consistent with those of the corresponding standards. Jayantha et al. reported precursor ions of *m*/*z* 397.40 and 383.40 for β-sitosterol and campesterol, respectively, along with product ions at *m*/*z* 134.9 and 161.2 (β-sitosterol) and *m*/*z* 147.1 and 161.1 (campesterol) [39]. Krauss et al. reported a precursor ion of *m*/*z* 288.2528 for lauroyl diethanolamide [40], while MassBank reference spectra (Accession ID: MSBNK-CASMI_2016-SM876702) contain product ions at *m*/*z* 106.0862 and 227.2005. These reports agree with the results obtained in this study, retention time, pre-cursor ion, and product ions of lauroyl diethanolamide, β-sitosterol, and campesterol in SP-EtOH-Ex were match. (Table 1). Therefore, the annotation confidence for lauroyl diethanolamide, β-sitosterol, and campesterol in SP-EtOH-Ex corresponds to Level 1 according to the criteria of Schymanski et al., indicating highly reliable identifications. In contrast, coniferyl aldehyde, mellein, and cycloartenol did not match the RTs or precursor ions of their authentic standards. For N-hexadecanoylpyrrolidine, values were similar for RTs and precursor ions, but inconsistent for product ions (Figure S3A). Gibberellin A3 and cinnamic acid were not detected (S/N < 3) under the same analytical conditions used during annotation.

Phytosterols are widely reported to possess anti-inflammatory properties [41]. It has been reported that sweet potatoes also contain plant sterols such as beta-sitosterol and campesterol [42,43,44]. Long-term high-dose intake of total phytosterols, including campesterol and β-sitosterol, has been associated with a modest reduction in coronary artery disease (CAD) risk [28]. Studies on rheumatoid arthritis (RA) have also suggested beneficial effects of sweet potato and phytosterols on chronic inflammatory diseases [45]. Lanlan Yuan et al. reported that campesterol exhibited a stronger inhibitory effect than β-sitosterol on the proliferation of LPS-stimulated RAW264.7 macrophages [46] and in human monocytic U937 cells, sitosterol, campesterol, and 7-keto-sitosterol decreased IL-8 secretion [47]. The mechanisms of β-sitosterol and campesterol have been reported to involve signaling pathways such as NF-κB, MAPK, and PPARγ [48,49,50,51]. In our previous study, we also reported that hydrophobic components derived from sweet potato storage roots attenuated LPS-induced inflammatory responses via activation of the Nrf2 pathway and subsequent suppression of NF-κB signaling [7]. The results of these previous studies are consistent with our findings that IL-6 production is suppressed by β-sitosterol and campesterol present in SP-EtOH-Ex (Figure 4). Beyond their cellular effects, phytosterols are well recognized for their nutritional benefits in humans. Clinical studies have demonstrated that daily intake of plant sterols lowers LDL-cholesterol by 5–15% by reducing intestinal cholesterol absorption [52,53]. Emerging evidence also suggests that phytosterols can modulate systemic inflammation and oxidative stress, and may improve glucose and lipid metabolism in individuals with metabolic syndrome, reinforcing their value as functional food ingredients [54,55]. The finding that the edible storage roots of the widely eaten yellow sweet potato can be a source of β-sitosterol and campesterol is thought to expand the future potential of sweet potatoes. Several limitations of this study should be acknowledged. First, the sweet potato cultivars, growing regions, and harvest periods analyzed were limited. Future studies are needed to explore various conditions, including those from various growing regions (e.g., Japan and Southeast Asia), various varieties, and different annual climatic conditions. Only ethanol was employed in the extraction method, and extraction conditions that might yield higher levels of phytosterols were not explored. It will be particularly important to explore efficient extraction and purification/separation conditions for industrialization. The analytical platform was restricted to LC-Q-TOF-MS; the inclusion of more advanced LC–MS/MS systems would likely enhance the confidence in compound identification. Anti-inflammatory assays were also conducted exclusively in mouse-derived macrophages, and whether similar effects occur in other cell types should be determined. In addition, the effectiveness of plant sterols has begun to be verified in humans, and the effectiveness of sweet potato-derived plant sterols needs to be verified through clinical trials.

## 4. Materials and Methods

### 4.1. Preparation of Sweet Potato Samples and Extracts

Sweet potato storage roots produced in Ibaraki or Chiba Prefecture, Japan, were washed with water and sliced into round pieces approximately 2–3 cm thick. The slices were freeze-dried using a freeze dryer (FDU-540; Tokyo Rika Kikai, Tokyo, Japan), and the dried material was pulverized to a fine powder using a food processor (MK-K62; Panasonic, Osaka, Japan). For extraction, 0.5 g of the powder was mixed with 25 mL of LC/MS-grade ethanol (Fujifilm Wako Pure Chemical, Osaka, Japan) and homogenized at 12,000 rpm for 2 min using a homogenizer (T 25 digital ULTRA-TURRAX; IKA-Werke GmbH & Co. KG, Staufen, Germany). The homogenate was centrifuged at 3000 rpm for 10 min using a KUBOTA 5922 centrifuge (Kubota Corporation, Osaka, Japan), and the resulting supernatant was collected. This extraction process was repeated four times, and all steps were performed at room temperature. The combined supernatants were evaporated to dryness with a rotary evaporator (N-1000; Tokyo Rika Kikai, Tokyo, Japan) and a diaphragm vacuum pump (DTC-41; ULVAC KIKO, Miyazaki, Japan), after which the residue was reconstituted in ethanol at a concentration of 10 mg/mL. The extract solution was then fractionated using an Agilent 1260 Infinity system (Agilent Technologies, Santa Clara, CA, USA) equipped with an ODS column (Cadenza CD-C18, 250 × 4.6 mm, particle size 3 µm; Imtakt Corporation, Kyoto, Japan). Chromatographic separation was performed at 40 °C with an injection volume of 30 µL. Solvent A (0.4% formic acid; Fujifilm Wako Pure Chemicals, Osaka, Japan) and solvent B (100% acetonitrile; Kanto Chemical, Tokyo, Japan) were used as mobile phases at a flow rate of 1 mL/min. The gradient was programmed from the initial condition (A:B = 93:7) to A:B = 60:40 at 33 min, followed by A:B = 0:100 at 40 min, and then maintained at A:B = 0:100 for 30 min. The fraction eluted between 51 and 60 min was collected (designated as SP-EtOH-Ex). The collected fraction was evaporated to dryness using a rotary evaporator and a diaphragm vacuum pump and then reconstituted in LC/MS-grade ethanol at a concentration of 1.0 mg/mL. Three independently prepared extracts (SP-EtOH-Ex 1–3) were stored at −80 °C until use.

### 4.2. Reagents

LPS (*Escherichia coli* O127) was purchased from Fujifilm Wako Pure Chemical Corporation (Osaka, Japan). The reference standards used in this study were procured as follows: lauroyl diethanolamide from Combi-Blocks (San Diego, CA, USA); β-sitosterol and campesterol from Tama Biochemical Co., Ltd. (Tokyo, Japan); N-hexadecanoylpyrrolidine from Angene (London, UK); coniferyl aldehyde from ChemScene (Monmouth Junction, NJ, USA); mellein and cycloartenol from MedChemExpress (Monmouth Junction, NJ, USA); gibberellin A3 from TargetMol Chemicals (Boston, MA, USA); and cinnamic acid from BLDpharm (Shanghai, China).

### 4.3. Analysis of SP-EtOH-Ex by LC-Q-TOF-MS

LC analyses were performed using an Agilent 1260 series system (Agilent Technologies, Santa Clara, CA, USA) equipped with either a Waters Atlantis dC18 column (150 × 2.1 mm, 3 µm; Waters Corporation, Milford, MA, USA) or a Waters Atlantis T3 column (150 × 2.1 mm, 3 µm; Waters Corporation). Mass spectrometric detection was carried out using an Agilent 6530 Q-TOF mass spectrometer (Agilent Technologies, Santa Clara, CA, USA) operated in positive-ion mode with electrospray ionization (ESI) or atmospheric pressure chemical ionization (APCI).

For ESI, the mobile phases consisted of acetonitrile (solvent A) and 0.1% formic acid in water (solvent B). The gradient program was as follows: 0–5 min, 15% A; 5–20 min, 15–55% A; 20–27 min, 55–90% A; 27–34 min, 90% A; 34–34.1 min, 90–15% A; and 34.1–50 min, 15% A. The flow rate was set to 0.2 mL/min. The MS parameters were as follows: Vcap, 3500 V; nebulizer pressure, 35 psi; fragmentor, 150 V; skimmer, 65 V; sheath-gas temperature, 375 °C; sheath-gas flow, 11.0 L/min; collision energy, 20 eV; and mass range, *m*/*z* 100–1700.

For APCI, the mobile phases consisted of methanol or methanol/isopropanol (IPA) (50:50, *v*/*v*) (solvent A) and either 0.1% formic acid in water or 10 mM ammonium formate (pH 6.2) in water (solvent B). The gradient was programmed as follows: 0–5 min, 15% A; 5–20 min, 15–55% A; 20–27 min, 55–90% A; 27–39 min, 90% A; 39–39.1 min, 90–15% A; and 39.1–55 min, 15% A. The flow rate was 0.6 mL/min when methanol was used as solvent A and 0.4 mL/min when methanol/IPA (50:50, *v*/*v*) was used. The column temperature was maintained at 45 °C, and the injection volume was 5 µL (Table S1). The MS parameters were as follows: Vcap, 3500 V; nebulizer pressure, 60 psi; fragmentor, 150 V; skimmer, 65 V; vaporizer temperature, 350 °C; gas flow, 5.0 L/min; collision energy, 20 eV; and mass range, *m*/*z* 100–1700. Each SP-EtOH-Ex 1–3 was analyzed in triplicate to ensure reproducibility of chromatographic measurements.

Both ESI and APCI data were acquired in data-dependent acquisition (DDA) mode.

### 4.4. Data Analysis for Annotating Unknown Compounds

Raw data were first processed using MS-DIAL (Ver. 5.3) [56]. The parameters were set as follows: MS1 tolerance, 0.01 Da; MS2 (product-ion) tolerance, 0.025 Da; minimum peak height, 1000; mass slice width, 0.1 Da; and sigma window, 0.5. The MS/MS database used for annotation was MSMS-Public_all-pos-VS19 (https://systemsomicslab.github.io/compms/msdial/main.html, accessed on 10 September 2024). Annotation of detected compounds was subsequently performed using MS-FINDER (Ver. 3.61). The parameters for MS-FINDER were set as follows: MS1 tolerance, 0.01 Da; MS2 tolerance, 0.025 Da; and mass range, *m*/*z* 0–2000. For element selection, C and H were specified as required elements and O, N, P, and S as optional elements. Annotation was conducted based on information from 23 integrated databases.

Next, FooDB [57] and KNApSAcK [58] were used to narrow down the annotated compounds and select candidate metabolites for identification with reference standards. In addition, MassBank Peak List Search (https://massbank.jp/MassBank/search, accessed on 22 January 2025) was employed to retrieve compounds whose MS2 spectra exhibited similarity to the experimentally obtained spectra.

### 4.5. Identification of Compounds by LC-Q-TOF-MS Using Reference Standards

The LC conditions (column, mobile phases, temperature, and injection volume) were identical to those used for compound annotation. Chromatographic separation was performed using a Waters Atlantis dC18 column for lauroyl diethanolamide and campesterol. A Waters Atlantis T3 column was used for all remaining standards. Ionization was conducted in positive-ion mode, using electrospray ionization (ESI) for lauroyl diethanolamide and N-hexadecanoylpyrrolidine and atmospheric pressure chemical ionization (APCI) for all remaining standards.

The mobile phases were as follows: acetonitrile (A) and 0.1% aqueous formic acid (B) for lauroyl diethanolamide and N-hexadecanoylpyrrolidine; methanol (A) and 0.1% aqueous formic acid (B) for coniferyl aldehyde and mellein; methanol/IPA (50:50, *v*/*v*) (A) and 0.1% aqueous formic acid (B) for gibberellin A3; methanol (A) only for campesterol; and methanol/IPA (50:50, *v*/*v*) (A) with 10 mM ammonium formate (pH 6.2) (B) for the remaining standards.

The gradient programs were as follows: for lauroyl diethanolamide and N-hexadecanoylpyrrolidine, 0–5 min: 15% A; 5–20 min: 15–55% A; 20–27 min: 55–90% A; 27–34 min: 90% A; 34–34.1 min: 90–15% A; and 34.1–50 min: 15% A. For coniferyl aldehyde, gibberellin A3, and cinnamic acid, 0–5 min: 15% A; 5–20 min: 15–55% A; 20–27 min: 55–90% A; 27–39 min: 90% A; 39–39.1 min: 90–15% A; and 39.1–55 min: 15% A. β-Sitosterol, campesterol, mellein, and cycloartenol were analyzed under isocratic conditions: 100% A from 0 to 5 min for campesterol, and 90% A from 0 to 10 min for β-sitosterol, mellein, and cycloartenol.

The flow rates were set as follows: 0.2 mL/min for lauroyl diethanolamide and N-hexadecanoylpyrrolidine; 0.4 mL/min for β-sitosterol, gibberellin A3, and cinnamic acid; 0.5 mL/min for coniferyl aldehyde, mellein, and cycloartenol; and 0.6 mL/min for campesterol (Table S2).

The mass ranges for MS and MS/MS acquisition were *m*/*z* 50–440 for lauroyl diethanolamide, *m*/*z* 50–550 for β-sitosterol and campesterol, *m*/*z* 100–1700 for gibberellin A3, *m*/*z* 50–1000 for cinnamic acid, and *m*/*z* 100–1100 for the other standards. All standards were analyzed using the targeted MS/MS (data-dependent acquisition; DDA) mode. Collision energies were set to 10 eV for lauroyl diethanolamide and mellein, 25 eV for β-sitosterol, and 20 eV for all other compounds.

### 4.6. Data Analysis for Confirming and Quantifying Unknown Compounds by Reference Standards

Data used for compound identification with reference standards were acquired using Agilent MassHunter Qualitative Analysis (B.07.00; Agilent Technologies, Santa Clara, CA, USA). For each target compound, the Find by Targeted MS/MS algorithm was used to extract the compound chromatogram (CC), retention time (RT), major product ions, and peak area.

### 4.7. Cell Culture and Stimulation Conditions

Mouse macrophage RAW264.7 cells were purchased from KAC (Kyoto, Japan). The cells were cultured in Dulbecco’s Modified Eagle Medium (DMEM; Sigma–Aldrich, St. Louis, MO, USA) supplemented with 10% deactivated fetal bovine serum (FBS; Biowest, Nuaillé, France) and penicillin–streptomycin (Gibco, Grand Island, NY, USA) at 37 °C in a humidified atmosphere containing 5% CO_2_.

### 4.8. Enzyme-Linked Immunosorbent Assay (ELISA)

RAW264.7 cells were seeded at a density of 1.0 × 10^4^ cells/well in 96-well plates and incubated for 24 h. SP-EtOH-Ex was added to the culture medium at a final concentration of 10 µg/mL, followed by incubation for 1 h, after which lipopolysaccharide (LPS) was added at a final concentration of 50 ng/mL. Each reference standard was treated in the same manner: the compound was added to the medium, incubated for 1 h, and subsequently stimulated with LPS (50 ng/mL). SP-EtOH-Ex and all reference standards were dissolved in LC/MS-grade ethanol. The same final concentration of ethanol was added to the control group to ensure that any effects observed were not attributable to the solvent. After 24 h of incubation, the culture supernatant was collected, and the concentration of IL-6 was measured using an ELISA MAX™ Deluxe Set Mouse IL-6 kit (BioLegend, San Diego, CA, USA).

### 4.9. Statistical Analysis

Statistical analysis was performed with GraphPad Prism 9 version 9.0.2 (GraphPad Software Inc., San Diego, CA, USA). One-way analysis of variance (ANOVA) was performed to test three or more groups, followed by Tukey’s post hoc test. The significance level was set at 5%.

## 5. Conclusions

In this study, β-sitosterol and campesterol were identified in the ethanol extract of sweet potato storage roots and were demonstrated to contribute to their anti-inflammatory activity. The edible portion of sweet potatoes holds promise as a raw material with anti-inflammatory properties.

## Figures and Tables

**Table 1:** The molecular formulae, ion species, theoretical values of precursor ions, RTs, measured values of precursor ions, mass errors, and product ions for the nine candidate compounds.

Annotated Compound According to MS-FINDER	Molecular Formula	Ion Species	Theoretical (*m*/*z*)	Reference Standard or SP-EtOH-Ex	Retention Time (min)	Experimental (*m*/*z*)	Error (ppm)	Product Ions	Identification Result
Lauroyl diethanolamide	C_16_H_33_NO_3_	[M+H]^+^	288.2533	Reference standard	25.94	288.25	6.94	106.0864 (100), 288.2513 (47), 88.0762 (33)	Yes
				SP-EtOH-Ex	25.90–25.96	288.2510–288.2531	0.69–7.98	106.0861 (100), 288.2510 (47), 88.0760 (34)	
β-sitosterol	C_29_H_50_O	[M-H_2_O+H]^+^	397.3829	Reference standard	4.745	397.3818	2.77	161.1327 (100), 81.0706 (77), 95.0865 (68)	Yes
				SP-EtOH-Ex	4.717–4.796	397.3820–397.3845	−4.03–2.26	161.1333 (100), 81.0706 (78), 95.0855 (71)	
Campesterol	C_28_H_48_O	[M-H_2_O+H]^+^	383.3672	Reference standard	2.672	383.3668	1.04	383.3668 (100), 161.1326 (85), 147.1173 (55)	Yes
				SP-EtOH-Ex	2.669–2.689	383.3623–383.3678	−1.57–6.26	383.3661 (100), 161.1329 (73), 147.1184 (62)	
N-Hexadecanoylpyrrolidine	C_20_H_39_NO	[M+H]^+^	310.3104	Reference standard	31.458	310.31	1.29	310.3100 (100), 311.3130 (23), 312.3168 (3)	No
				SP-EtOH-Ex	31.18–31.20	310.3087–310.3108	−1.29–5.48	109.0649 (100), 109.1001 (81), 135.1164 (80)	
Coniferyl aldehyde	C_10_H_10_O_3_	[M+H]^+^	179.0703	Reference standard	12.711	179.0707	−2.23	119.0493 (100), 147.0441 (60), 146.0364 (35)	No
				SP-EtOH-Ex	33.435	179.1535	−464.62	119.0493 (100), 147.0440 (83), 148.0462 (11)	
Mellein	C_10_H_10_O_3_	[M+H]^+^	179.0703	Reference standard	1.069	179.0694	5.03	161.0594 (100), 179.0694 (70), 133.0642 (26)	No
				SP-EtOH-Ex	7.284–7.522	179.0636–179.0668	19.55–37.42	147.0435 (100), 148.0470 (12), 119.0493 (11)	
Gibberellin A3	C_19_H_22_O_6_	[M+H]^+^	347.1489	Reference standard	N.A.	N.A.	N.A.	N.A.	No
				SP-EtOH-Ex	–	–	–	–	
Cinnamic acid	C_9_H_8_O_2_	[M-H_2_O+H]^+^	131.0491	Reference standard	N.A.	N.A.	N.A.	N.A.	No
				SP-EtOH-Ex	–	–	–	–	
Cycloartenol	C_30_H_50_O	[M-H_2_O+H]^+^	409.3829	Reference standard	3.57	409.3833	−0.98	109.1015 (100), 191.1799 (80), 121.1015 (71)	No
				SP-EtOH-Ex	4.201–4.282	409.3779–409.3861	−7.82–12.21	191.1802 (100), 409.3799 (63), 109.1012 (60)	

For SP-EtOH-Ex, RT, precursor ion measured value, and mass error. For product ions, one representative ion was selected from SP-EtOH-Ex 1, 2, or 3 and listed. The brackets for the product ions represent the relative intensity ratio, with the intensity of the strongest product ion set to 100. SP-EtOH-Ex: ethanol extract of sweet potato storage roots; RT: retention time; N.A.: not applicable.

**Table 2:** Quantitative results of SP-EtOH-Ex for lauroyl diethanolamide, β-sitosterol, and campesterol.

Compound	Concentration of SP-EtOH-Ex		
	1	2	3
Lauroyl diethanolamide (ng/mL)	8.113	9.260	4.568
β-sitosterol (µg/mL)	4.850	2.839	2.527
Campesterol (ng/mL)	93.63	75.74	N.A.

SP-EtOH-Ex: ethanol extract of sweet potato storage roots; N.A.: not applicable.

## Data Availability

The data presented in this study are available on reasonable request from the corresponding author.

## Supplementary Materials

The following supporting information can be downloaded at: https://www.mdpi.com/article/10.3390/molecules31030456/s1, Figure S1. Precursor ions and product ions of compounds annotated in MS-FINDER but not meeting Schymanski et al.’s identification criteria based on reference standards; Figure S2. CC values for SP-EtOH-Ex annotated as N-hexadecanoylpyrrolidine, coniferyl aldehyde, mellein, and cycloaltenol, respectively, and CC values for corresponding standards; Figure S3. Product ions for SP-EtOH-Ex annotated as N-hexadecanoylpyrrolidine, coniferyl aldehyde, mellein, and cycloartenol, respectively, and product ions for corresponding standards; Table S1. Measurement conditions for DDA analysis; Table S2. Measurement conditions for identification using reference standards; Table S3. Ranking of candidate compounds with potential anti-inflammatory effects contained in SP-EtOH-Ex among compounds annotated by MS-FINDER in MS-FINDER and MassBank; Table S4. The molecular formulae, ion species, precursor ion theoretical values, RTs, measured values for precursor ions, mass errors, and product ions for the nine candidate compounds.
