# Supplementary material for: Identification of Active Anti-Inflammatory Compounds in Sweet Potato Storage Roots Extracted with Ethanol via LC-Q-TOF-MS
Source: Molecules. 2026 Jan 28;31(3):456. doi: 10.3390/molecules31030456 (PMC12898583; doi:10.3390/molecules31030456)
Supplement: Supplementary file 1 [file molecules-31-00456-s001.zip › molecules-4098667-supplementary.pdf]

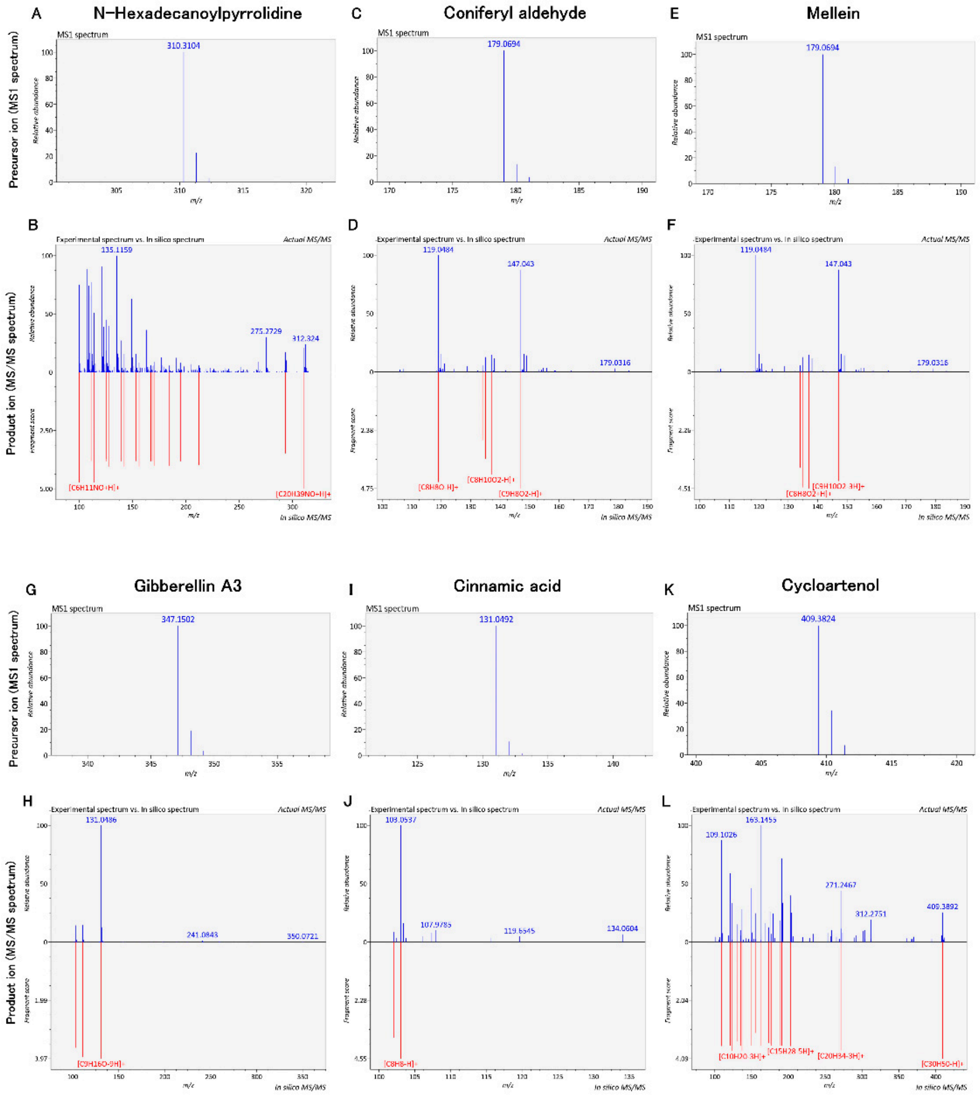

Supplementary Figure S1. Precursor ions and product ions of compounds annotated in MS-FINDER but not meeting Schymanski et al.'s identification criteria based on reference standards. The compound annotated as N-Hexadecanoylpyrrolidine (A,B), Coniferyl aldehyde (C, D), Mellein(E, F), Gibberellin A3 (G, H), Cinnamic acid(I, J), Cycloartenol(K)(L).

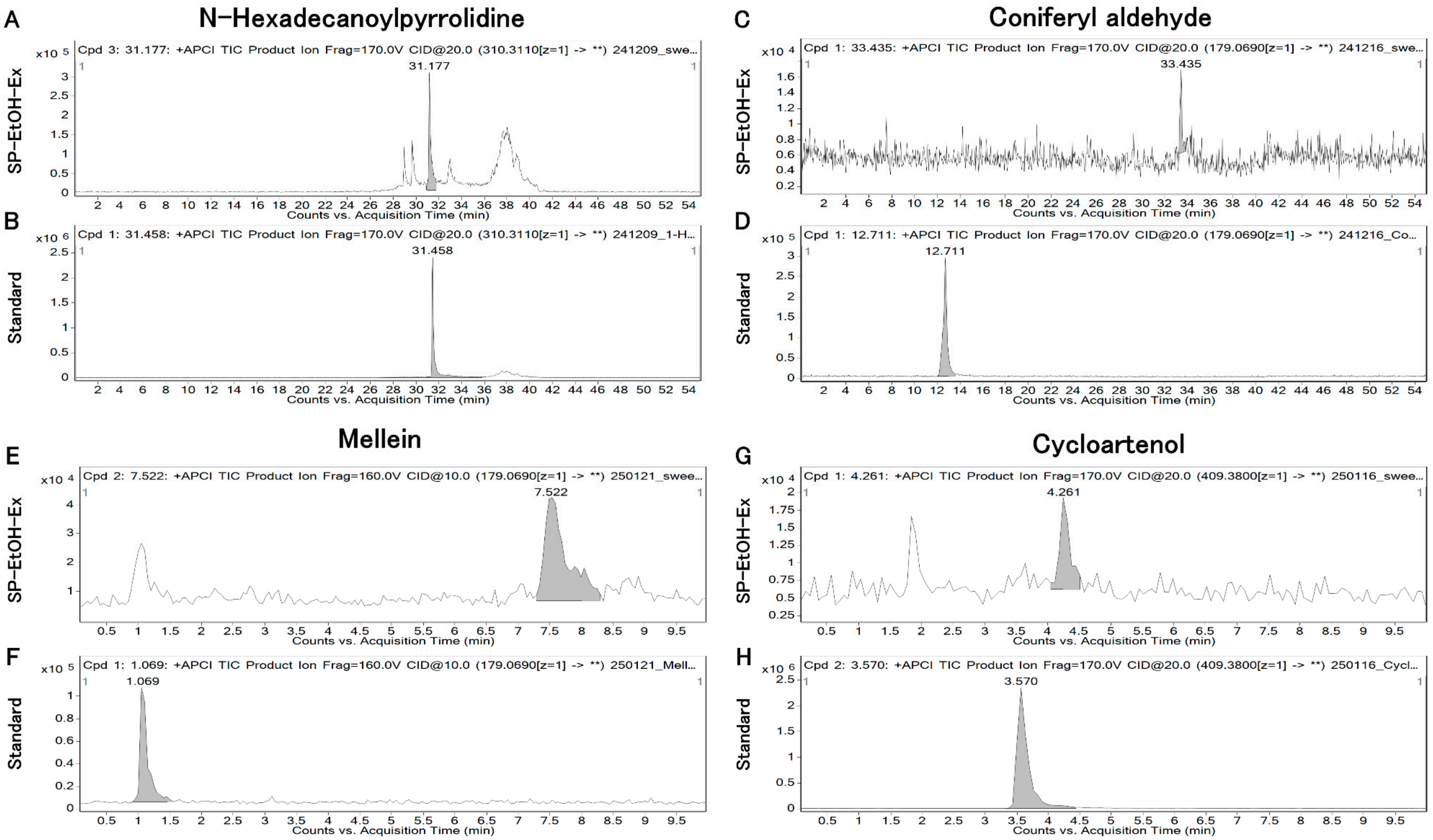

Supplementary Figure S2. CC values for SP-EtOH-Ex annotated as N-hexadecanoylpyrrolidine, coniferyl aldehyde, mellein, and cycloaltenol, respectively, and CC values for corresponding standards. For Gibberellin A3 and Cinnamic acid, no data is available because the standards were not detected when analyzed under the same conditions as the Auto MSMS measurements: The compound annotated as N-Hexadecanoylpyrrolidine (A, B), Coniferyl aldehyde(C, D), Mellein(E, F), Cycloartenol(G)(H).

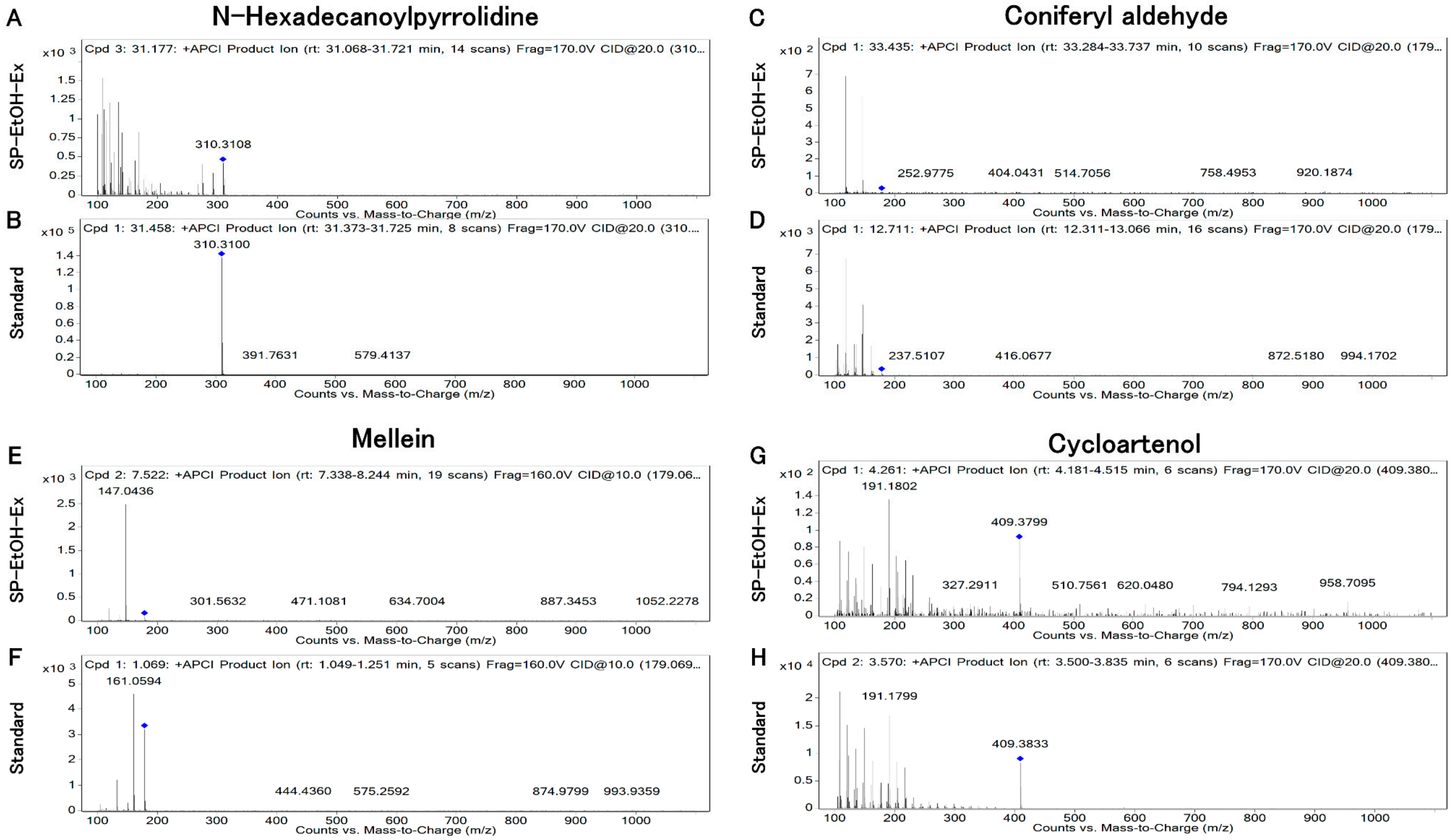

Supplementary Figure S3. Product ions for SP-EtOH-Ex annotated as N-hexadecanoylpyrrolidine, coniferyl aldehyde, mellein, and cycloartenol, respectively, and product ions for corresponding standards. For Gibberellin A3 and Cinnamic acid, no data is available because the standards were not detected when analyzed under the same conditions as the Auto MSMS measurements. : The compound annotated as N-Hexadecanoylpyrrolidine (A, B), Coniferyl aldehyde(C, D), Mellein (E, F), Cycloartenol(G, H).

Supplementary Table S1. Measurement conditions for DDA analysis (MeCN; Acetonitrile, MeOH; Methanol, IPA; Isopropanol, 0.1 % FA; 0.1% formic acid aqueous solution, 10 mM AF; 10 mM ammonium formate solution)

Ion mode	Mobile phaze A	LC columns	LC Flow rate (mL/min)	Mobile phaze B	Gradient	Annotated compound by MS-FINDER
ESI	MeCN	Atlantis dC18	0.2	0.1% FA	0–5 min, 15% A; 5–20 min, 15–55% A; 20–27 min, 55–90% A; 27–34 min, 90% A; 34–34.1 min, 90–15% A; 34.1–50 min, 15% A	Lauroyl diethanolamide
ESI	MeCN	Atlantis T3	0.2	0.1% FA	0–5 min, 15% A; 5–20 min, 15–55% A; 20–27 min, 55–90% A; 27–34 min, 90% A; 34–34.1 min, 90–15% A; 34.1–50 min, 15% A	N-Hexadecanoylpyrrolidine
APCI	MeOH	Atlantis T3	0.6	0.1% FA	0–5 min, 15% A; 5–20 min, 15–55% A; 20–27 min, 55–90% A; 27–39 min, 90% A; 39–39.1 min, 90–15% A; 39.1–55 min, 15% A	Coniferyl aldehyde, Mellein
APCI	MeOH/IPA (50:50 v/v)	Atlantis dC18	0.4	0.1% FA	0–5 min, 15% A; 5–20 min, 15–55% A; 20–27 min, 55–90% A; 27–39 min, 90% A; 39–39.1 min, 90–15% A; 39.1–55 min, 15% A	Campesterol
APCI	MeOH/IPA (50:50 v/v)	Atlantis T3	0.4	0.1% FA	0–5 min, 15% A; 5–20 min, 15–55% A; 20–27 min, 55–90% A; 27–39 min, 90% A; 39–39.1 min, 90–15% A; 39.1–55 min, 15% A	Gibberellin A3
APCI	MeOH/IPA (50:50 v/v)	Atlantis T3	0.4	10 mM AF	0–5 min, 15% A; 5–20 min, 15–55% A; 20–27 min, 55–90% A; 27–39 min, 90% A; 39–39.1 min, 90–15% A; 39.1–55 min, 15% A	β-sitosterol, Cinnamic acid, Cycloartenol

Supplementary Table S2. Measurement conditions for identification using reference standards

Mobile phaze B	Mobile phaze A	Compound	Gradient	LC columns	Ion mode	LC Flow rate (mL/min)
0.1% FA	MeCN	Lauroyl diethanolamide	0–5 min, 15% A; 5–20 min, 15–55% A; 20–27 min, 55–90% A; 27–34 min, 90% A; 34–34.1 min, 90–15% A; 34.1–50 min, 15% A	Atlantis dC18	ESI	0.2
10 mM AF	MeOH/IPA (50:50 v/v)	β-sitosterol	0–10 min, 90% A	Atlantis T3	APCI	0.4
–	MeOH	Campesterol	0–5 min, 100% A	Atlantis dC18	APCI	0.6
0.1% FA	MeCN	N-Hexadecanoylpyrrolidine	0–5 min, 15% A; 5–20 min, 15–55% A; 20–27 min, 55–90% A; 27–34 min, 90% A; 34–34.1 min, 90–15% A; 34.1–50 min, 15% A	Atlantis T3	ESI	0.2
0.1% FA	MeOH	Coniferyl aldehyde	0–5 min, 15% A; 5–20 min, 15–55% A; 20–27 min, 55–90% A; 27–39 min, 90% A; 39–39.1 min, 90–15% A; 39.1–55 min, 15% A	Atlantis T3	APCI	0.5
0.1% FA	MeOH	Mellein	0–10 min, 90% A	Atlantis T3	APCI	0.5
0.1% FA	MeOH/IPA (50:50 v/v)	Gibberellin A3	0–5 min, 15% A; 5–20 min, 15–55% A; 20–27 min, 55–90% A; 27–39 min, 90% A; 39–39.1 min, 90–15% A; 39.1–55 min, 15% A	Atlantis T3	APCI	0.4
10 mM AF	MeOH/IPA (50:50 v/v)	Cinnamic acid	0–5 min, 15% A; 5–20 min, 15–55% A; 20–27 min, 55–90% A; 27–39 min, 90% A; 39–39.1 min, 90–15% A; 39.1–55 min, 15% A	Atlantis T3	APCI	0.4
10 mM AF	MeOH/IPA (50:50 v/v)	Cycloartenol	0–10 min, 90% A	Atlantis T3	APCI	0.5

Supplementary Table S3. Ranking of candidate compounds with potential anti-inflammatory effects contained in SP-–EtOH-–Ex among compounds annotated by MS-FINDER in MS-FINDER and Mass-Bank

Annotated compound	MS-FINDER			MassBank		
	Rank	Number of Candidates	Score	Rank	Number of Candidates	Score
Lauroyl diethanolamide	1–2	3	6.64–7.84	1	281–500 <	0.5971–0.7504
β-sitosterol	1	78	7.47–8.11	1	500 <	0.6152–0.7513
Campesterol	1	49	6.66–7.88	1	500 <	0.6883–0.7793
N-Hexadecanoylpyrrolidine	1–2	3	6.42–6.80	—	—	—
Coniferyl aldehyde	1	100 <	6.92–8.54	4–139	500 <	0.1219–0.5368
Mellein	2–40	100 <	6.55–7.85	—	500 <	—
Gibberellin A3	1	100 <	7.46–7.48	—	500 <	—
Cinnamic acid	1	43	5.97–7.77	—	102–420	—
Cycloartenol	3–8	100 <	6.23–6.87	—	500 <	—

Supplementary Table S4. The molecular formulae, ion species, precursor ion theoretical values, RTs, measured values for precursor ions, mass errors, and product ions for the nine candidate compounds. For SP-EtOH-Ex, RT, precursor ion measured value, and mass error and each sample was measured three times. The brackets in the product ions represent the relative intensity ratio, with the intensity of the strongest product ion set to 100.

Annotated compound by MS-FINDER	Molecular formula	Ion Species	Theortectical (*m/z*)	CE (eV)	Reference standard or SP-EtOH-Ex	RT (min)	Experimental (*m/z*)	Error (ppm)	Fragment ions	Identification results
Lauroyl diethanolamide	$C_{16}H_{33}NO_3$	$[M+H]^+$	288.2533	10	Reference standard	25.94	288.2513	6.94	106.0864 (100), 288.2513 (47), 88.0762 (33)	Yes
					SP-EtOH-Ex 1-1	25.96	288.2510	7.98	106.0861 (100), 288.2510 (47), 88.0760 (34)	
					SP-EtOH-Ex 1-2	25.95	288.2511	7.63	106.0862 (100), 288.2511 (46), 88.0759 (35)	
					SP-EtOH-Ex 1-3	25.95	288.2524	3.12	106.0862 (100), 288.2524 (44), 88.0762 (33)	
					SP-EtOH-Ex 2-1	25.90	288.2519	4.86	106.0860 (100), 288.2519 (48), 88.0759 (35)	
					SP-EtOH-Ex 2-2	25.90	288.2518	5.20	106.0860 (100), 288.2518 (48), 88.0754 (36)	

					SP-EtOH-Ex 2-3	25.90	288.2519	4.86	106.0861 (100), 288.2519 (42), 88.0759 (34)	
					SP-EtOH-Ex 3-1	25.90	288.2527	2.08	106.0868 (100), 288.2527 (48), 88.0758 (31)	
					SP-EtOH-Ex 3-2	25.89	288.2531	0.69	106.0860 (100), 288.2531 (42), 88.0758 (31)	
					SP-EtOH-Ex 3-3	25.89	288.2525	2.78	106.0861 (100), 288.2525 (43), 88.0760 (31)	
β-sitosterol	$C_{29}H_{50}O$	$[M-H_2O+H]^+$	397.3829	25	Reference standard	4.745	397.3818	2.77	161.1327 (100), 81.0706 (77), 95.0865 (68)	Yes
					SP-EtOH-Ex 1-1	4.796	397.3834	-1.26	161.1320 (100), 81.0705 (94), 95.0861 (88)	
					SP-EtOH-Ex 1-2	4.762	397.3830	-0.25	161.1333 (100), 81.0706 (78), 95.0855 (71)	
					SP-EtOH-Ex 1-3	4.737	397.3839	-2.52	161.1333 (100), 81.0704 (66), 95.0861 (68)	

					SP-EtOH-Ex 2-1	4.759	397.3845	-4.03	161.1324 (100), 81.0707 (81), 95.0860 (65)	
					SP-EtOH-Ex 2-2	4.767	397.3823	1.51	161.1323 (100), 81.0705 (80), 95.0859 (61)	
					SP-EtOH-Ex 2-3	4.718	397.3834	-1.26	161.1329 (100), 81.0708 (96), 95.0868 (97)	
					SP-EtOH-Ex 3-1	4.724	397.3820	2.26	161.1332 (100), 81.0707 (81), 95.0862 (67)	
					SP-EtOH-Ex 3-2	4.717	397.3836	-1.76	161.1331 (100), 81.0708 (80), 95.0860 (83)	
					SP-EtOH-Ex 3-3	4.742	397.3838	-2.26	161.1331 (100), 81.0701 (88), 95.0859 (63)	
Campesterol	$C_{28}H_{48}O$	$[M-H_2O+H]^+$	383.3672	20	Reference standard	2.672	383.3668	1.04	383.3668 (100), 161.1326 (85), 147.1173 (55)	Yes
					SP-EtOH-Ex 1-1	2.675	383.3661	2.87	383.3661 (100), 161.1329 (73), 147.1184 (62)	

					SP-EtOH-Ex 1-2	2.689	383.3667	1.30	383.3667 (100), 161.1320 (98), 147.1175 (79)	
					SP-EtOH-Ex 1-3	2.677	383.3654	4.70	383.3654 (100), 161.1320 (83), 147.1142 (50)	
					SP-EtOH-Ex 2-1	2.668	383.3678	-1.57	383.3678 (100), 161.1323 (94), 147.1169 (63)	
					SP-EtOH-Ex 2-2	2.669	383.3660	3.13	383.3660 (100), 161.1326 (90), 147.1159 (56)	
					SP-EtOH-Ex 2-3	2.670	383.3648	6.26	383.3648 (100), 161.1332 (70), 147.1182 (60)	
					SP-EtOH-Ex 3-1	N.A.	N.A.	N.A.	N.A.	
					SP-EtOH-Ex 3-2	N.A.	N.A.	N.A.	N.A.	
					SP-EtOH-Ex 3-3	N.A.	N.A.	N.A.	N.A.	
N-Hexadecanoylpyrrolidine	$C_{20}H_{39}NO$	$[M+H]^+$	310.3104	20	Reference standard	31.46	310.3100	1.29	310.3100 (100), 311.3130 (23), 312.3168 (3)	No
					SP-EtOH-Ex 1-1	N.A.	N.A.	N.A.	N.A.	
					SP-EtOH-Ex 1-2	N.A.	N.A.	N.A.	N.A.	
					SP-EtOH-Ex 1-3	N.A.	N.A.	N.A.	N.A.	

					SP-EtOH-Ex 2-1	31.18	310.3108	-1.29	109.0649 (100), 109.1001 (81), 135.1164 (80)	
					SP-EtOH-Ex 2-2	31.18	310.3101	0.97	109.0652 (100), 135.1164 (96), 121.1009 (83)	
					SP-EtOH-Ex 2-3	31.17	310.3092	3.87	109.0650 (100), 109.1007 (92), 135.1164 (89)	
					SP-EtOH-Ex 3-1	31.20	310.3087	5.48	109.0648 (100), 169.0851 (60), 141.0903 (54)	
					SP-EtOH-Ex 3-2	31.19	310.3103	0.32	109.0648 (100), 109.0994 (59), 121.1004 (57)	
					SP-EtOH-Ex 3-3	31.19	310.3098	1.93	109.0647 (100), 169.0854 (57), 141.0902 (56)	
Coniferyl aldehyde	$C_{10}H_{10}O_3$	$[M+H]^+$	179.0703	20	Reference standard	12.711	179.0707	-2.23	119.0493 (100), 147.0441 (60), 146.0364 (35)	No
					SP-EtOH-Ex 1-1	33.435	179.1535	-464.62	119.0493 (100), 147.0440 (83), 148.0462 (11)	
					SP-EtOH-Ex 1-2	—	—	—	—	

					SP-EtOH-Ex 1-3	—	—	—	—	
					SP-EtOH-Ex 2-1	—	—	—	—	
					SP-EtOH-Ex 2-2	—	—	—	—	
					SP-EtOH-Ex 2-3	—	—	—	—	
					SP-EtOH-Ex 3-1	—	—	—	—	
					SP-EtOH-Ex 3-2	—	—	—	—	
					SP-EtOH-Ex 3-3	—	—	—	—	
Mellein	$C_{10}H_{10}O_3$	$[M+H]^+$	179.0703	20	Reference standard	1.069	179.0694	5.03	161.0594 (100), 179.0694 (70), 133.0642 (26)	No
					SP-EtOH-Ex 1-1	7.522	179.0668	19.55	147.0436 (100), 148.0468 (13), 119.0488 (10)	
					SP-EtOH-Ex 1-2	—	—	—	—	
					SP-EtOH-Ex 1-3	—	—	—	—	
					SP-EtOH-Ex 2-1	7.284	179.0636	37.42	147.0435 (100), 148.0470 (12), 119.0493 (11)	
					SP-EtOH-Ex 2-2	—	—	—	—	
					SP-EtOH-Ex 2-3	—	—	—	—	
					SP-EtOH-Ex 3-1	—	—	—	—	

					SP-EtOH-Ex 3-2	—	—	—	—	
					SP-EtOH-Ex 3-3	—	—	—	—	
Gibberellin A3	$C_{19}H_{22}O_6$	$[M+H]^+$	347.1489	20	Reference standard	N.A.	N.A.	N.A.	N.A.	No
					SP-EtOH-Ex 1-1	—	—	—	—	
					SP-EtOH-Ex 1-2	—	—	—	—	
					SP-EtOH-Ex 1-3	—	—	—	—	
					SP-EtOH-Ex 2-1	—	—	—	—	
					SP-EtOH-Ex 2-2	—	—	—	—	
					SP-EtOH-Ex 2-3	—	—	—	—	
					SP-EtOH-Ex 3-1	—	—	—	—	
					SP-EtOH-Ex 3-2	—	—	—	—	
					SP-EtOH-Ex 3-3	—	—	—	—	
Cinnamic acid	$C_9H_8O_2$	$[M-H_2O+H]^+$	131.0491	20	Reference standard	N.A.	N.A.	N.A.	N.A.	No
					SP-EtOH-Ex 1-1	—	—	—	—	
					SP-EtOH-Ex 1-2	—	—	—	—	
					SP-EtOH-Ex 1-3	—	—	—	—	
					SP-EtOH-Ex 2-1	—	—	—	—	
					SP-EtOH-Ex 2-2	—	—	—	—	

					SP-EtOH-Ex 2-3	—	—	—	—	
					SP-EtOH-Ex 3-1	—	—	—	—	
					SP-EtOH-Ex 3-2	—	—	—	—	
					SP-EtOH-Ex 3-3	—	—	—	—	
Cycloartenol	$C_{30}H_{50}O$	$[M-H_2O+H]^+$	409.3829	20	Reference standard	3.570	409.3833	-0.98	109.1015 (100), 191.1799 (80), 121.1015 (71)	No
					SP-EtOH-Ex 1-1	4.261	409.3799	7.33	191.1802 (100), 109.1012 (64), 409.3799 (64)	
					SP-EtOH-Ex 1-2	4.250	409.3810	4.64	109.1011 (100), 191.1751 (91), 409.3810 (79)	
					SP-EtOH-Ex 1-3	4.282	409.3861	-7.82	409.3861 (100), 109.1004 (93), 135.1177 (83)	
					SP-EtOH-Ex 2-1	4.204	409.3800	7.08	121.1002 (100), 191.1781 (100), 149.1307 (81)	
					SP-EtOH-Ex 2-2	4.201	409.3840	-2.69	149.1312 (100), 409.3840 (100), 135.1189 (81)	

					SP-EtOH-Ex 2-3	4.249	409.3779	12.21	163.1461 (100), 191.1803 (88), 123.1175 (73)	
					SP-EtOH-Ex 3-1	4.252	409.3837	-1.95	135.1156 (100), 109.1022 (96), 409.3837 (73)	
					SP-EtOH-Ex 3-2	4.281	409.3842	-3.18	123.1161 (100), 191.1819 (85), 409.3842 (70)	
					SP-EtOH-Ex 3-3	4.255	409.3780	11.97	135.1158 (100), 109.1015 (84), 149.1320 (78)	
